# MicroRNA-196b-5p promotes malignant progression of colorectal cancer by targeting ING5

**DOI:** 10.1186/s12935-020-01200-3

**Published:** 2020-04-10

**Authors:** He Xin, Chuanzhuo Wang, Yuan Chi, Zhaoyu Liu

**Affiliations:** Department of Radiology, Shengjing Hospital of China Medical University, 36 Sanhao Street, Shenyang, 110004 People’s Republic of China

**Keywords:** Colorectal cancer, MiR-196b-5p, ING5

## Abstract

**Background:**

miR-196b-5p expression is deregulated in many malignant tumors. Although miR-196b-5p has been implicated in the malignant transformation of colorectal cancer, its role in this specific type of cancer has not been fully explored. Thus, the present study was aimed to examine the cellular function of miR-196b-5p and its role in malignant biological behavior in colorectal cancer.

**Methods:**

miR-196b-5p expression was measured in colorectal cancer tissues and cell lines using quantitative real-time PCR. Cell counting kit-8 (CCK-8) assay and Transwell assay were used to detect proliferation, migration, and invasion in cell lines, whereas flow cytometry was applied to study apoptosis. Western blot analysis was performed to measure the protein levels. Dual luciferase reporter assay was used to investigate the interaction between miR-196b-5p and ING5. Tumor formation was evaluated in mice.

**Results:**

MiR-196b-5p was abundantly expressed in colorectal cancer tissues and cell lines, whereas ING5 was expressed at low levels. MiR-196b-5p was successfully overexpressed or knocked down in colorectal cancer cells. We found that miR-196b-5p overexpression significantly accelerated the proliferation, cell cycle, migration and invasion, while inhibited cell apoptosis in colorectal cancer cells. However, miR-196b-5p inhibitor showed the opposite effects. Moreover, ING5 overexpression or knockdown was successfully performed in colorectal cancer cells. ING5 overexpression suppressed proliferation, migration, invasion, the phosphorylation of PI3K, Akt as well as MEK, and promoted cell apoptosis, which could be reversed by ING5 knockdown. Additionally, ING5 was identified as a target of miR-196b-5p through bioinformatics analysis and a luciferase activity assay. Furthermore, ING5 knockdown could attenuate the decrease in proliferation, migration, invasion, and the protein levels of p-PI3K, p-Akt, and p-MEK, which were induced by miRNA-196b-5p inhibitor. Besides, miR-196b-5p knockdown inhibited tumor growth, whereas ING5 knockdown elevated it in vivo.

**Conclusions:**

In conclusion, miR-196b-5p promotes cell proliferation, migration, invasion, and inhibits apoptosis in colorectal cancer by targeting ING5.

## Background

Colorectal cancer (CRC) is one of the most common malignancies worldwide, with more than 1 million cases diagnosed every year [1, 2]. The morbidity of CRC ranks second among the lethal tumors [3]. The high mortality of CRC is closely correlated with tumor metastasis, particularly liver metastasis [4, 5]. There is approximately 10–15% overall 5-year survival rate among patients with metastatic CRC [6, 7]. Although surgical excision has been applied in CRC treatment [8], the results of surgical therapies are unsatisfactory. Therefore, it is necessary to explore therapeutic targets to improve the effectiveness of CRC treatment.

MicroRNAs (miRNAs) are single-stranded, endogenous and short non-coding RNA molecules [9, 10]. MiRNAs regulate gene expression through targeting 3′-untranslated region (3′-UTR) of their target genes, causing mRNA degradation or translation repression [11, 12]. Increasing evidences have shown that miRNAs participate in tumorigenesis and tumor development via regulating a variety of signaling pathways (cell cycle, proliferation, angiogenesis, differentiation, invasion and metastasis) [13–15]. Moreover, miRNAs have been found to act as a tumor suppressor or a tumor promoter (“oncomiR”) in cancers [16, 17], including CRC. For example, Sun et al. demonstrated that miR-708 inhibited cell proliferation, metastasis and induced cell apoptosis in CRC [18]. He et al. discovered that miR-150 played a suppressive role in cell viability and proliferation in CRC cells [19]. Conversely, miR-410 was verified to promote CRC cell proliferation, invasion, migration and repressed apoptosis [20]. Interestingly, miR-196b-5p has been reported to be related to CRC development [21], indicating its involvement in the CRC tumorigenesis.

Inhibitor of growth 5 (ING5) is identified as Class II tumor suppressor and involved in promoting DNA repair, causing apoptosis and chromatin remodeling, which depend on the formation of histone acetyl transferase (HAT) complexes [22–24]. Previous study revealed that ING5 could interact with p53, and thereby inhibiting cell growth and inducing apoptosis [25, 26]. Moreover, ING5 was reported to inhibit the initiation, promotion and development of tumors [27]. Accumulating evidence has showed that ING5 overexpression represses colony formation efficiency and induces apoptosis in CRC cells [28]. Bioinformation analysis has predicted that ING5 gene is a potential target of miR-196b-5p. Thus, we speculate that miR-196b-5p may take part in the progression of CRC via targeting ING5.

In the current study, miR-196b-5p level was measured in CRC tissues and adjacent tissues. Then, we analyzed the impact of miR-196b-5p on CRC cell proliferation, migration, invasion and apoptosis in vitro and in vivo. In vitro, miR-196b-5p mimics and inhibitor were conducted in CRC cells. In vivo, the xenograft models were established in nude mice. Functional analyses revealed that miR-196b-5p could act as a tumor promoter (“oncomiR”) in CRC through targeting ING5. The data suggests that miR-196b-5p may be a candidate biomarker for CRC treatment.

## Materials and methods

### Clinical sample collection

CRC tissue samples (n = 5) and adjacent tissue samples (n = 5) were collected in CRC surgical excision in Shengjing Hospital of China Medical University between January 2018 and January 2019. These samples were quickly transferred into liquid nitrogen for preservation. Each patient had been given informed consent. The study was approved by the Shengjing Hospital ethics committee and according to Declaration of Helsinki.

### Cell culture and transfection

The normal human colorectal cell line (FHC) and human CRC cell lines (SW480 and HCT116) were purchased from the Cell Resource Center, Chinese Academy of Sciences Committee (Shanghai, China). FHC and HCT116 cells were cultured in DMEM (Gibco, USA) supplemented with 10% fetal bovine serum (BI, USA) at 37 °C in 5% CO_2_. SW480 cells were cultured in L15 medium (Sigma, USA) supplemented with 10% fetal bovine serum (BI, USA) at 37 °C in 5% CO_2_. The miR-196b-5p (MIMAT0001080) mimics, inhibitor and negative control (miR-196b-5p NC mimics/miR-196b-5p NC inhibitor) were obtained from GenePharma Co. Ltd. (Shanghai, China). The CRC cell lines (SW480 and HCT116) (4 × 10^5^ cells/well) were transfected with miR-196b-5p NC mimics, miR-196b-5p mimics, miR-196b-5p NC inhibitor or miR-196b-5p inhibitor with Lipofectamine 2000 (Invitrogen, Carlsbad, CA, USA) for 48 h. The sequences are listed in Table 1. Additionally, to overexpress ING5 (Gene ID: 84289), the plasmid (pcDNA3.1-ING5) was constructed by the Chinese Academy of Sciences (Changchun, China). The full-length CDS of ING5 was cloned into pcDNA3.1 plasmid between *BamHI* and *XhoI* restriction enzyme sites. The plasmid knocking down ING5 (ING5 shRNA) and control shRNA (shRNA NC) were purchased from Sigma-Aldrich (St. Louis, MO, USA). The shRNA sequences targeting ING5 was cloned into plasmid. The sequence of ING5 shRNA was listed in Table 1. SW480 and HCT116 cells were transfected with 2 μg ING5 overexpression plasmid (pcDNA3.1-ING5) or control plasmid (pcDNA3.1) with Lipofectamine 2000 (Invitrogen, Carlsbad, CA, USA) and exposed to Geneticin G418 to select ING5 overexpression (ING5 (+)) or ING5 overexpression negative control (ING5 (+) NC) CRC cells. SW480 and HCT116 cells were transfected with 2 μg ING5 shRNA or shRNA NC with Lipofectamine 2000 (Invitrogen, Carlsbad, CA, USA) and exposed to Geneticin G418 to select ING5 knockdown (ING5 (−)) or ING5 knockdown negative control (ING5 (−) NC) CRC cells. Similarly, miR-196b-5p inhibitor and ING5 (−) co-transfection was conducted by the same way in SW480 and HCT116 cells.

**Table 1:** The sequences were used in this study

Name	Sequences (5′–3′)
miR-196b-5p	Stem-loop RT primer: GCGCGTGAGCAGGCTGGAGAAATTAACCACGCGCCCCAAC
	F: GCGTAGGTAGTTTCCTG
	R: GAGCAGGCTGGAGAA
miR-196a-1	Stem-loop RT primer: GCGCGTGAGCAGGCTGGAGAAATTAACCACGCGCTCGGGTG
	F: CGCAACAACATTAAACC
	R: GAGCAGGCTGGAGAA
miR-196a-2	Stem-loop RT primer: GCGCGTGAGCAGGCTGGAGAAATTAACCACGCGCCTCAGG
	F: CGGCAACAAGAAACTG
	R: GAGCAGGCTGGAGAA
ING5	F: GCACAAAGGAGGGTCTGA
	R: TGGGTTTCGTGGTAAGGT
U6	F: GCTTCGGCAGCACATATACT
	R: GAGCAGGCTGGAGAA
β-actin	F: CTTAGTTGCGTTACACCCTTTCTTG
	R: CTGTCACCTTCACCGTTCCAGTTT
miR-196b-5p mimics	TAGGTAGTTTCCTGTTGTTGGG
miR-196b-5p inhibitor	CCCAACAACAGGAAACTACCTA
miR-196b-5p NC mimics	TTCTCCGAACGTGTCACGT
miR-196b-5p NC inhibitor	TTCTCCGAACGTGTCACGT
ING5 shRNA	F: CAAGGAATACAGTGACGACAA
	R: TTGTCGTCACTGTATTCCTTG

### Quantitative real-time PCR

Quantitative real-time PCR was performed based on a previously reported method [29]. In short, the total RNA of the aforementioned tissues or cells was isolated by TRIzol (Invitrogen) following the manufacturer’s instructions. The obtained RNA was transcribed into the relevant cDNA by using microRNA-specific stem-loop RT primer or relevant primer. Afterwards, quantitative real-time PCR was implemented to using Two Step SYBR^®^ Primer Script TM RT-PCR Kit (Takara Bio, Inc., China). The primers of miR-196b-5p, miR-196a-1, miR-196a-2 and ING5 were listed in Table 1. U6 and β-actin were the internal controls. Relative expression levels were calculated using the 2^‒ΔΔCt^ method.

### Western blot analysis

Total proteins were extracted by ice-cold-RIPA lysis buffer and protein concentrations were quantified using a BCA kit (Pierce, Rockford, IL, USA). The samples were separated by 8–10% SDS-PAGE gel (Beyotime, China) and then transferred onto PVDF membranes. After blocking with 5% BSA at room temperature for 2 h, the membranes were incubated with primary antibodies overnight at 4 °C. Subsequently, the horseradish peroxidase (HRP)-labeled secondary antibody (Proteintech Group, Inc.) was used to cover the membranes for 50 min at room temperature. The blots were developed using enhanced chemiluminescence. The primary antibodies were as follows: ING5 (Proteintech Group, Inc., Rosemont, IL, USA), PI3K, p-PI3K, AKT, p-AKT, MEK, and p-MEK (Cell Signaling Technology, Danvers, MA, USA), and GAPDH (Proteintech Group, Inc.). GAPDH was used as the loading control.

### Proliferation assay

A Cell Counting Kit-8 (CCK-8) (Thermo Fisher Scientific, USA) was used to assess cell viability according to the manufacturer’s instructions. Briefly, cells were seeded in 96‐well culture plates at a density of 5 × 10^4^ cells/200 μL/well. After transfection, the cells were grown for 72 h at 37 °C and CCK-8 reagent (10 μl/well) was added to the culture medium. After incubation for 2 h, the optical density of the solution was measured at 450 nm using a microplate reader.

### Cell cycle analysis

After the aforementioned transfection, the adherent cells were harvested and then fixed with 500 μl of 70% cold ethanol for 2 h. 100 μl of RNAse was added to the cells and incubated for 30 min at 37 °C. Then, the cells were incubated with 400 μl of PI at 4 °C for 30 min in the dark. The cell cycle was detected with flow cytometer (Calibur, BD Biosciences, USA) and the data was analyzed using ModFit LT.

### Cell migration and invasion assays

Cell migration and invasion were analyzed using Transwell assay (pore size: 8 µm) in the 24-well plates as previously described with appropriate improvement [30]. For the migration assay, 5 × 10^4^ cells were suspended in serum‐free medium and plated in upper chambers (Corning Costar, New York, NY, USA). The lower chambers were filled with 30% FBS medium. For the invasion assay, the upper chamber was coated with Matrigel (BD Bioscience, San Jose, CA, USA) according to the manufacturer’s protocols and then 5 × 10^4^ cells in serum‐free culture medium were added. The lower chambers were filled with 30% FBS medium. Finally, the migration and invasion cells stained with 0.1% crystal violet were counted under a microscope at 100× magnification, respectively.

### Apoptosis assay

Cell apoptosis was determined using a 7AAD Apoptosis Detection Kit I (BD Biosciences, San Jose, CA, USA) based on the manufacturer’s protocol. Transfected cells were cultured in serum-free medium for 24 h. Then, cells were collected and washed with cold PBS. Cells were resuspended in 1× binding buffer and subsequently stained with PE and 7AAD in the dark for 15 min. Apoptotic cells were analyzed using a fluorescence-activated cell-sorting (FACS) flow cytometer (BD Biosciences).

### Luciferase reporter assay

Targetscan (http://www.targetscan.org), a bioinformatics analysis website predicting microRNA targets in mammals, was used to predict the potential targeting relationship between ING5 and miR-196b-5p. To identify the relationship between miR-196b-5p and ING5, luciferase reporter assay was conducted. The sequences of wild-type ING5 3′-UTR containing the binding site of miR-196b-5p were inserted into the p-MIR luciferase reporter vector (Ambion) and named with ING5 3′-UTR Wt. The sequences of mutant type ING5 3′-UTR excluding the binding site of miR-196b-5p were inserted into the p-MIR luciferase reporter vector (Ambion) and named with ING5 3′-UTR Mut. Then, HEK 293T cells were co-transfected with ING5 3′-UTR Wt or ING5 3′-UTR Mut and miR-196b-5p NC mimics or miR-196b-5p mimics for 48 h using 3.75 μl Lipofectamine 3000 (Invitrogen). Finally, cells were washed with PBS and lysed, and luciferase activity was measured with a dual luciferase assay kit (Promega). The sequences of ING5 3′-UTR Wt and ING5 3′-UTR Mut were listed in Table 2.

**Table 2:** The sequences of ING5 3′-UTR Wt and ING5 3′-UTR Mut were used in this study

Name	Sequences (5′–3′)
ING5 3′-UTR Wt	GAGGAGCTGTGTGCCCGGATCCGAGGAGCAAGTTAATCTGTCCCTTCATTCGTGTCGCAATATTTCCCTTCCTTTTA**AAACTACCT**TGTTCGGTTGATACTTAGTAACTCCGTGGCCAGTTGAAGCGCTGGATGTTTCCTAGAACAAGAACCACCAAAGCCTGTTCGCACAGAAGGGCGACCTTGCAGGGACTCGCCGCCGCGACCTCAGTGTGGCTTTTACAGGACTCCCCCCGAGCATCAGCAGGGACCCCGGCGGACGTGGGCGGGCGCGCGTGAGCTCGGGCTGCCCGGCCGGGCGTGCGGGCGGGGACATGGTAACCTGGTCCACGGAGGGCGGCCGCCACCCTCGCGT
ING5 3′-UTR Mut	GAGGAGCTGTGTGCCCGGATCCGAGGAGCAAGTTAATCTGTCCCTTCATTCGTGTCGCAATATTTCCCTTCCTTTTA**CCGACCTTA**TGTTCGGTTGATACTTAGTAACTCCGTGGCCAGTTGAAGCGCTGGATGTTTCCTAGAACAAGAACCACCAAAGCCTGTTCGCACAGAAGGGCGACCTTGCAGGGACTCGCCGCCGCGACCTCAGTGTGGCTTTTACAGGACTCCCCCCGAGCATCAGCAGGGACCCCGGCGGACGTGGGCGGGCGCGCGTGAGCTCGGGCTGCCCGGCCGGGCGTGCGGGCGGGGACATGGTAACCTGGTCCACGGAGGGCGGCCGCCACCCTCGCGT

he bold text indicated the potential binding sites

### Xenograft models

Female BALB/c nude mice (6-8 weeks old) were housed in a specific pathogen-free (SPF) facility under a 12-h/12-h light/dark cycle. The animal work was performed according to the Guideline for the Care and Use of Laboratory Animals and approved by the Shengjing Hospital ethics committee.

The mice were randomly divided into five groups: (A) Control (mice received SW480 cells/HCT116 cells); (B) miR-196b-5p NC inhibitor (mice received SW480 cells/HCT116 cells transfected with miR-196b-5p NC inhibitor); (C) miR-196b-5p inhibitor (mice received SW480 cells/HCT116 cells transfected with miR-196b-5p inhibitor); (D) miR-196b-5p inhibitor + ING5(−)-NC (mice received SW480 cells/HCT116 cells co-transfected with miR-196b-5p inhibitor and ING5 NC shRNA); (E) miR-196b-5p inhibitor + ING5(−) (mice received SW480 cells/HCT116 cells co-transfected with miR-196b-5p inhibitor and ING5 shRNA). In short, 1 × 10^6^ cells SW480 cells/HCT116 cells were uniformly suspended in PBS at a final concentration of 1 × 10^7^ cells/ml after transfection, which procedures were consistent with “Cell culture and transfection” section. Then, 0.1 ml (1 × 10^6^ cells) was subcutaneously injected into BALB/c nude mice. After 7 days, tumor growth was monitored and the tumor size was recorded every 4 days. The tumor volume was calculated as follows: length × width × width × 0.5.

### Statistical analysis

Data were expressed as the means ± standard deviations (SD). Statistical analysis between two groups was performed with two-tailed paried or non-paired Student’s t-test. Comparisons among more than two groups were conducted using one-way analysis of variance (ANOVA) followed by Tukey’s test. A *p* value of < 0.05 was considered statistically significant.

## Results

### Expression of miR-196b-5p and ING5 in CRC tissues and cells

**Fig. 1:**
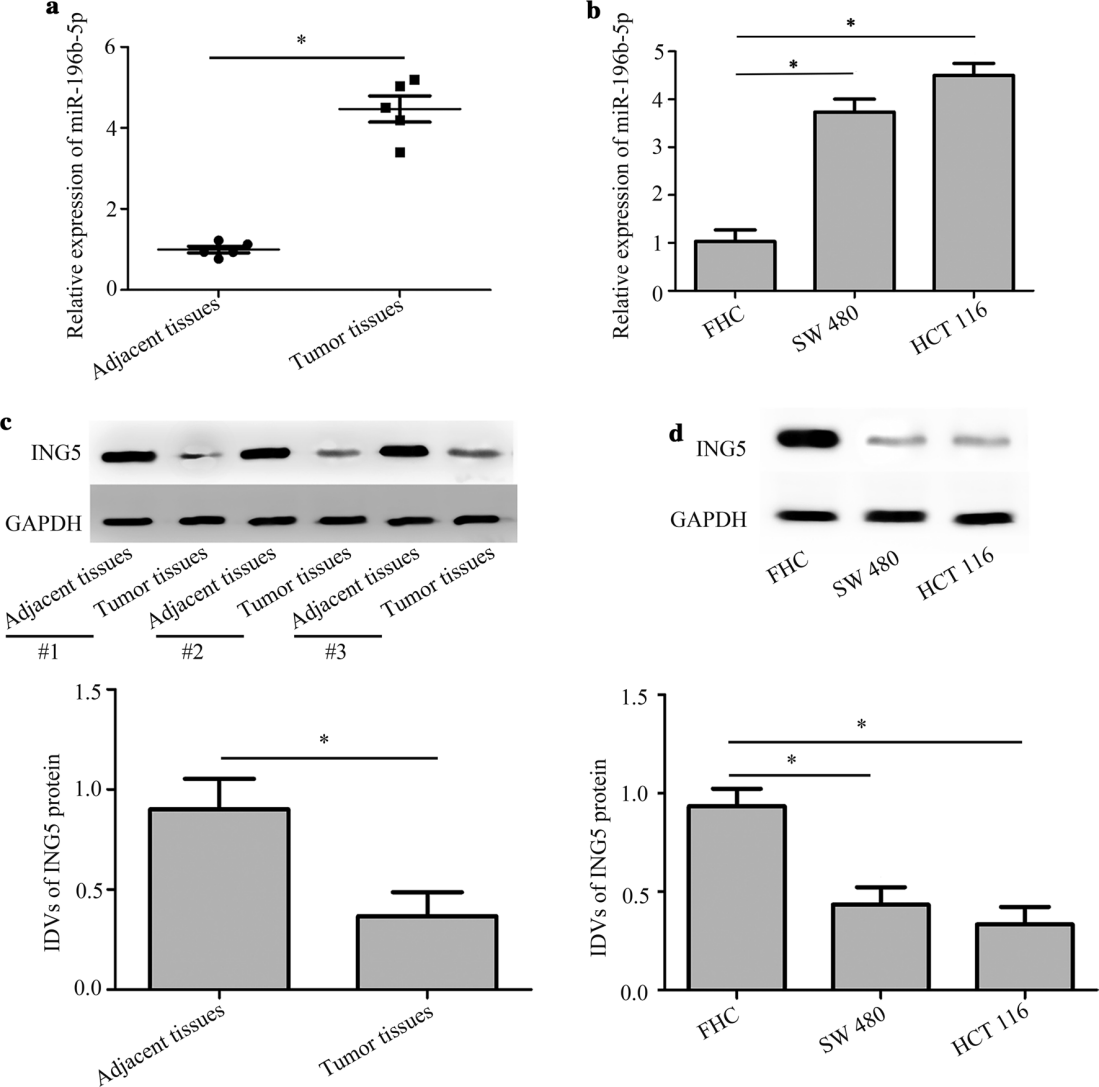
Expression of miR-196b-5p and ING5 in CRC tissues and cells. **a** Quantitative real-time PCR detected miR-196b-5p level in CRC tissues and adjacent tissues. n = 5. **b** Quantitative real-time PCR measured miR-196b-5p level in FHC cell line and CRC cell lines (SW480 and HCT116). n = 3. **c** ING5 protein level was detected in CRC tissues and adjacent tissues using western blot analysis. n = 3. **d** ING5 protein level was examined in FHC cell line and CRC cell lines (SW480 and HCT116) via western blot analysis. n = 3. *P < 0.05. *CRC* colorectal cancer, *ING5* inhibitor of growth 5

We first measured miR-196b-5p expression level in CRC tissues using quantitative real-time PCR and found that miR-196b-5p level was increased in CRC tissues compared with adjacent tissues (Fig. 1a). Moreover, quantitative real-time PCR showed that miR-196b-5p level was higher in CRC cells (SW480 and HCT116) than in FHC cells (Fig. 1b). Furthermore, western blot analysis demonstrated that ING5 protein level was lower in CRC tissues than in adjacent tissues (Fig. 1c). In addition, there was a decrease of ING5 protein level in SW480 and HCT116 cells compared with FHC cells (Fig. 1d). These results indicated that miR-196b-5p level was elevated while ING5 level was decreased in CRC tissues and cells.

### Effects of miR-196b-5p on cell proliferation, apoptosis, migration and invasion

**Fig. 2:**
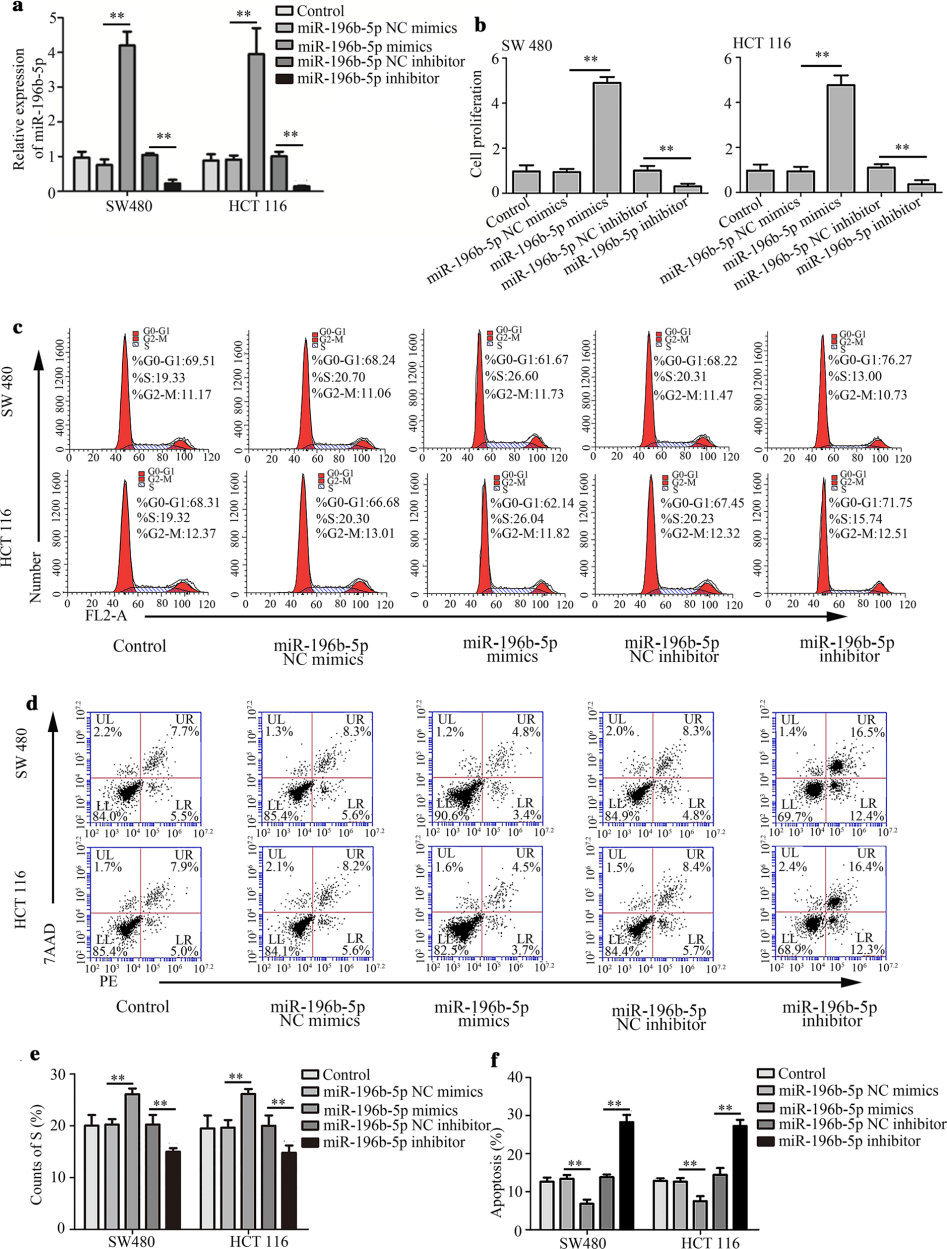
Effects of miR-196b-5p on CRC cell proliferation and apoptosis. **a** The level of miR-196b-5p was measured with quantitative real-time PCR. **b** CCK-8 assay was used to determine the proliferation. **c**–**f** Flow cytometry analysis detected cell cycle and apoptosis. n = 3. **P < 0.01. *CRC* colorectal cancer, *NC* negative control

**Fig. 3:**
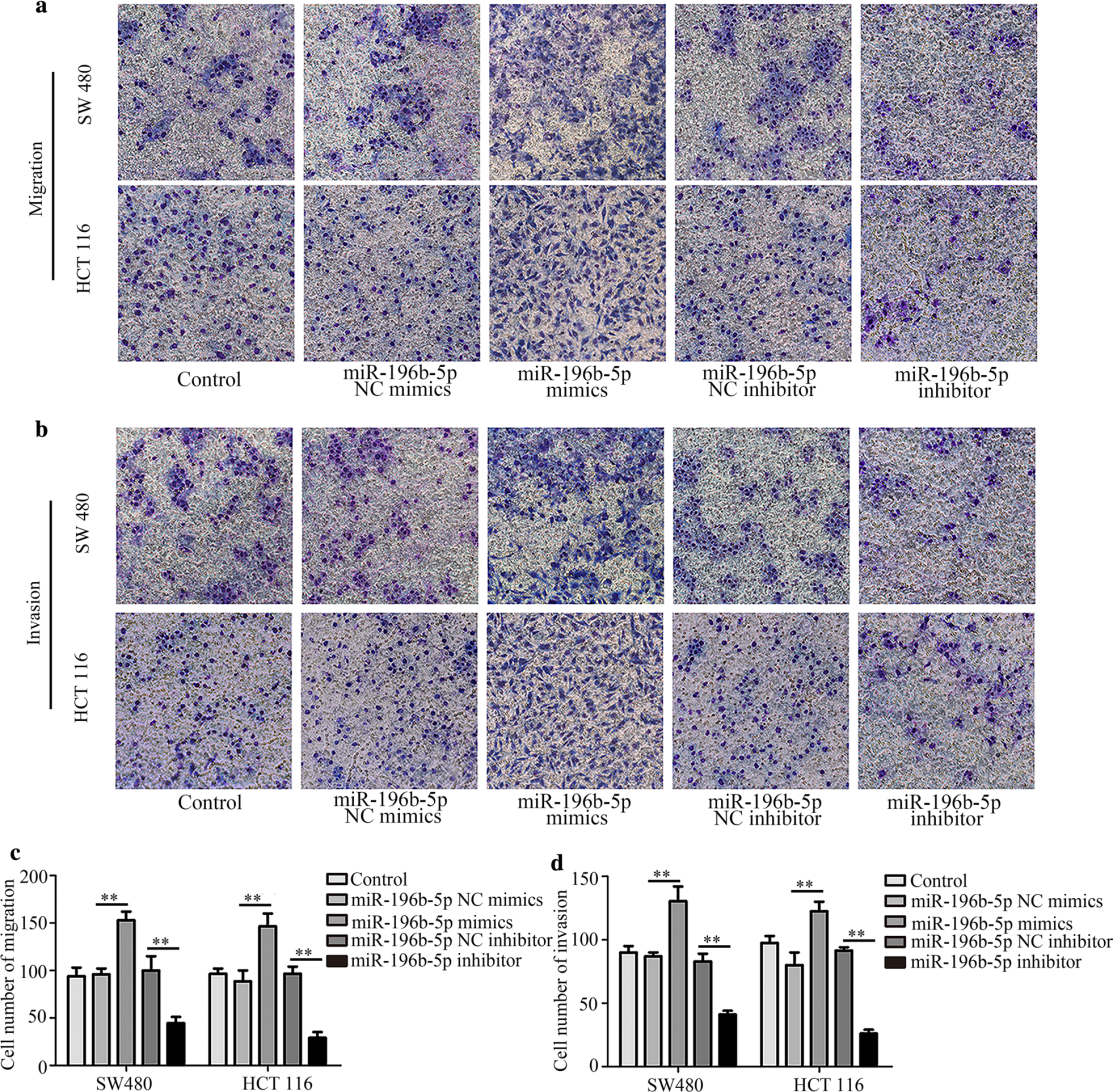
Effects of miR-196b-5p on CRC cell migration and invasion. **a**–**d** Transwell assay examined cell migration and invasion. Scale bar, 20 μm. n = 3. **P < 0.01. *CRC* colorectal cancer, *NC* negative control

MiR-196b-5p was overexpressed or silenced in SW480 and HCT116 cells to investigate the effect of miR-196b-5p expression on CRC progression. The quantitative real-time PCR revealed that miR-196b-5p level was increased in CRC cells with miR-196b-5p mimics, whereas miR-196b-5p level was decreased in CRC cells with miR-196b-5p inhibitor (Fig. 2a), indicating that CRC cells were successfully transfected with miR-196b-5p mimics or inhibitor. Moreover, miR-196b-5p mimics and miR-196b-5p inhibitor showed no effect on the level of miR-196a-1 and miR-196a-2 (Additional file 1: Fig. S1A–D), suggesting that miR-196b-5p mimics and miR-196b-5p inhibitor did not interfere and inhibit other miR-196 family members. Additionally, CCK-8 assay demonstrated that miR-196b-5p mimics increased proliferation in CRC cells, whereas miR-196b-5p inhibitor exhibited opposite effects (Fig. 2b). Flow cytometry analysis proved that miR-196b-5p mimics caused acceleration of cell cycle and suppression of cell apoptosis, whereas miR-196b-5p inhibitor reduced cell cycle and promoted cell apoptosis (Fig. 2c–f). Further, up-regulation of migration and invasion was induced by miR-196b-5p mimics and down-regulation of migration and invasion was caused by miR-196b-5p inhibitor in CRC cells using Transwell assays (Fig. 3a–d). These findings suggested that miR-196b-5p promoted cell proliferation, migration, invasion and suppressed cell apoptosis in CRC cells.

### Effects of ING5 on CRC cell proliferation, apoptosis, migration, invasion and on protein levels

**Fig. 4:**
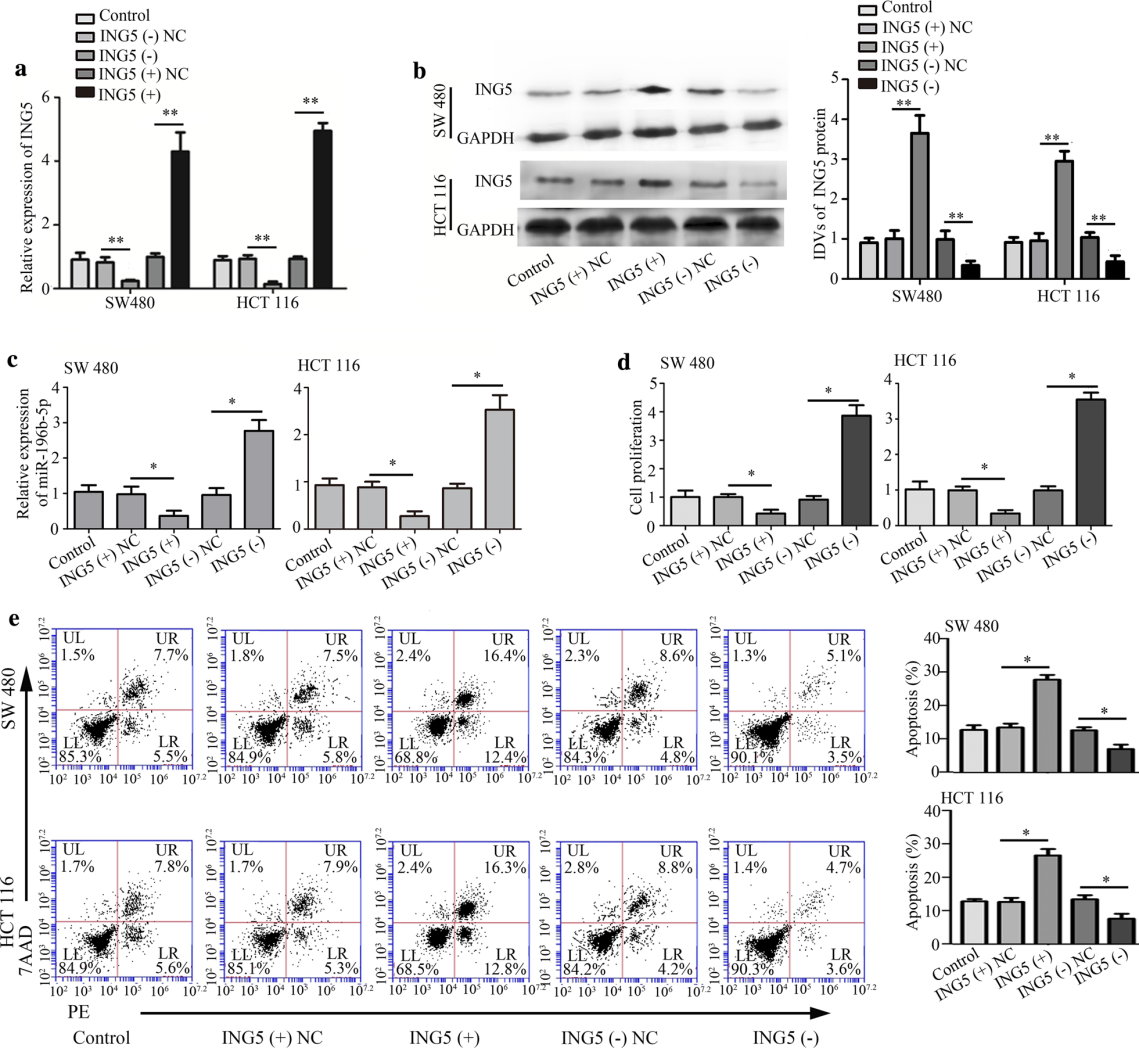
Effects of ING5 on CRC cell proliferation and apoptosis. **a**, **b** ING5 level was measured using quantitative real-time PCR and western blot analysis. **c** Quantitative real-time PCR was used to detect the level of miR-196b-5p. **d** Cell proliferation was measured through CCK-8 assay. **e** Flow cytometry analysis was used to examine cell apoptosis. n = 3. *P < 0.05. **P < 0.01. *CRC* colorectal cancer, *ING5* inhibitor of growth 5, *ING5 (+)* ING5 overexpression, *ING5 (−)* ING5 knockdown, *NC* negative control

**Fig. 5:**
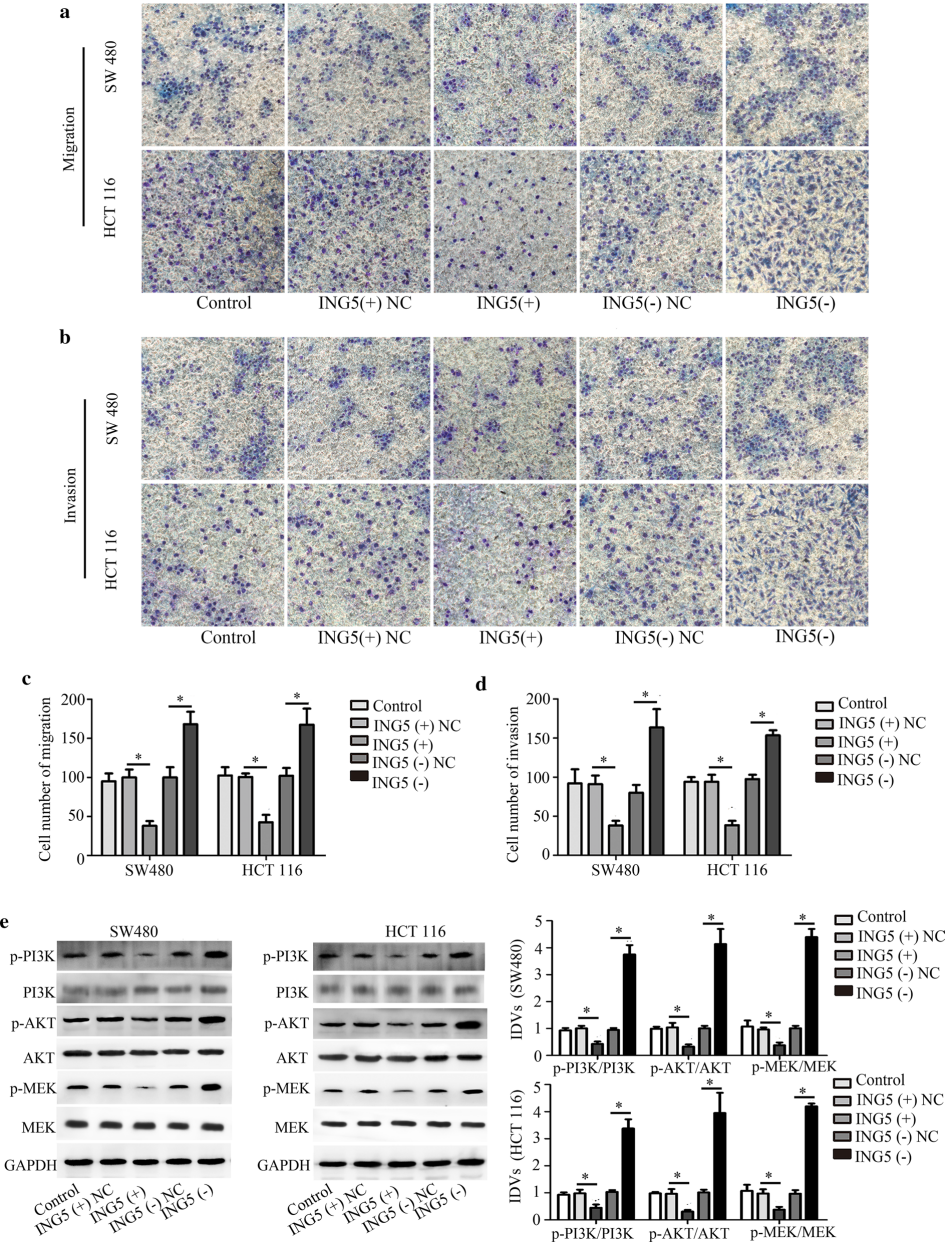
Effects of ING5 on CRC cell migration, invasion and protein levels. **a**–**d** Transwell assays were performed to detect cell migration and invasion. Scale bar, 20 μm. **e** Western blot analysis measured the PI3K, p-PI3K, Akt, p-Akt, MEK and p-MEK protein levels. n = 3. *P < 0.05. *CRC* colorectal cancer, *ING5* inhibitor of growth 5, *ING5 (+)* ING5 overexpression, *ING5 (−)* ING5 knockdown, *NC* negative control

We investigated the effects of ING5 on CRC cell properties through ING5 overexpression and knockdown. Quantitative real-time PCR and western blot analysis showed that ING5 level was increased in CRC cells (SW480 and HCT116) with ING5 overexpression, whereas decreased with ING5 knockdown in CRC cells (Fig. 4a, b), suggesting that ING5 overexpression and knockdown were successfully conducted. Additionally, quantitative real-time PCR demonstrated that miR-196b-5p level was decreased in CRC cells with ING5 overexpression and it was elevated in CRC cells with ING5 knockdown (Fig. 4c). However, ING5 overexpression and knockdown had no impact on the level of miR-196a-1 and miR-196a-2 (Additional file 2: Fig. S2A–D). CCK-8 assay revealed that cell proliferation was inhibited by ING5 overexpression while enhanced by ING5 knockdown in CRC cells (Fig. 4d). Moreover, flow cytometry analysis showed that ING5 overexpression promoted cell apoptosis and ING5 knockdown suppressed cell apoptosis (Fig. 4e). Transwell assays verified that ING5 overexpression played a suppressive role in cell migration and invasion, whereas ING5 knockdown increased cell migration and invasion (Fig. 5a–d). Further, western blot analysis showed that the protein levels of p-PI3K, p-Akt, and p-MEK were inhibited by ING5 overexpression, whereas these protein levels were enhanced by ING5 knockdown (Fig. 5e). The data implied that ING5 played a suppressive role in cell progression of CRC.

### ING5 is a target of miR-196b-5p

**Fig. 6:**
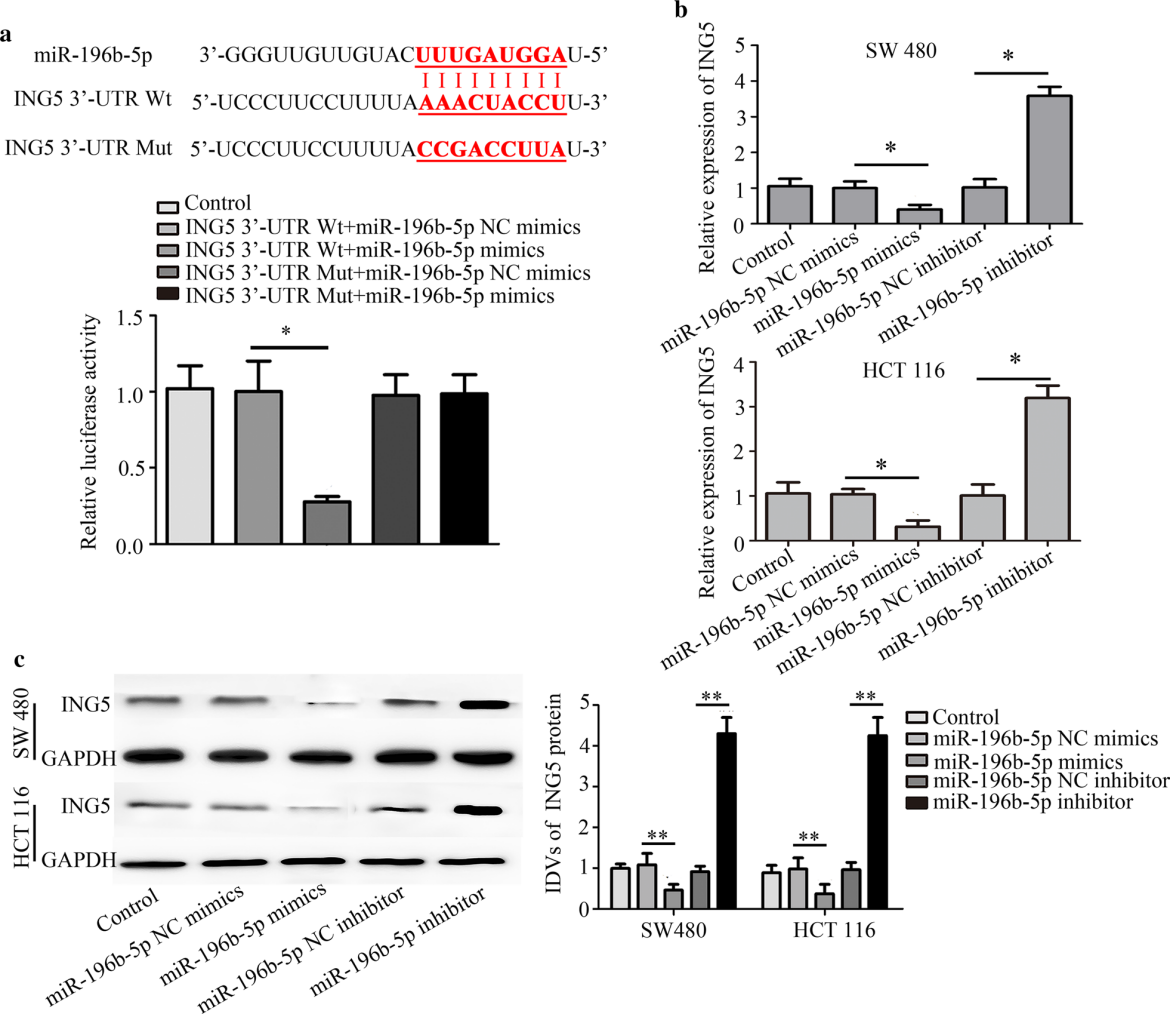
ING5 is a target of miR-196b-5p. **a** ING5 was predicted to be a target of miR-196b-5p using Targetscan (http://www.targetscan.org) analysis, and a dual luciferase reporter assay verified the relationship between miR-196b-5p and ING5. **b**, **c** Quantitative real-time PCR and western blot analysis measured the ING5 level. n = 3. *P < 0.05. **P < 0.01. *CRC* colorectal cancer, *ING5* inhibitor of growth 5, *NC* negative control

To explore the mechanism of miR-196b-5p in progression of CRC, we predicted the target gene of miR-196b-5p using Targetscan. The results predicted that ING5 was a target of miR-196b-5p (Fig. 6a). Moreover, the binding activity of miR-196b-5p on the ING5 3′-UTR was verified through a dual luciferase reporter assay. Results showed that miR-196b-5p mimics decreased the relative luciferase activity in HEK 293T cells co-transfected with miR-196b-5p mimics and ING5 3′-UTR Wt. However, the relative luciferase activity had no effect in HEK 293T cells co-transfected with miR-196b-5p mimics and ING5 3′-UTR Mut (Fig. 6a). Quantitative real-time PCR and western blot analysis proved that miR-196b-5p mimics decreased the ING5 expression and miR-196b-5p inhibitor increased ING5 expression in CRC cells (Fig. 6b, c). These results suggested that miR-196b-5p targeted ING5 expression via binding to its 3′-UTR.

### ING5 knockdown mediates the effects of miR-196b-5p inhibitor on CRC cell proliferation, apoptosis, migration, invasion and protein levels

**Fig. 7:**
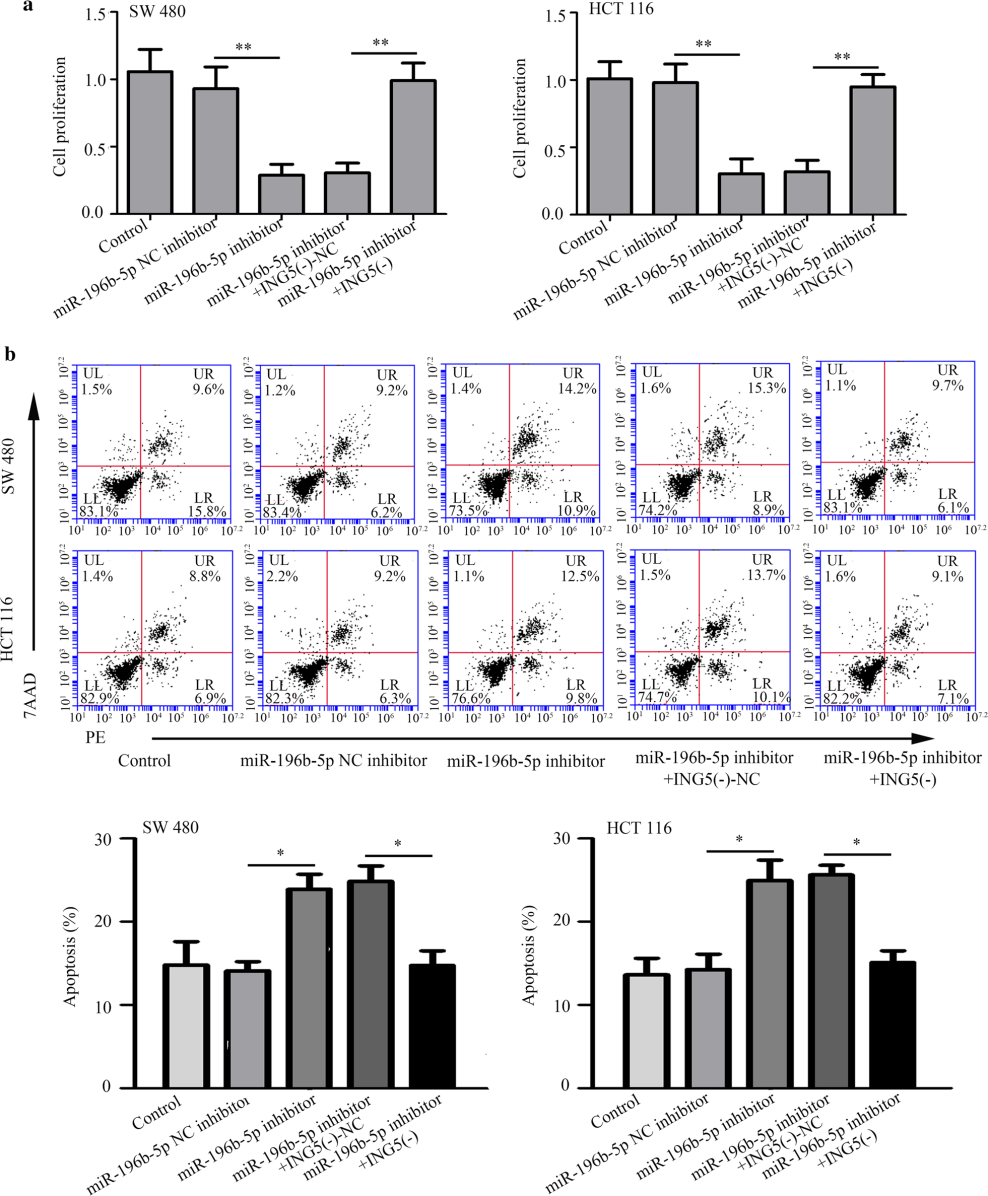
ING5 knockdown mediates the effects of miR-196b-5p inhibitor on CRC cell proliferation and apoptosis. **a** CCK-8 assay was conducted to measure cell proliferation. **b** Cell apoptosis was examined via flow cytometry analysis. n = 3. *P < 0.05. **P < 0.01. *CRC* colorectal cancer, *ING5* inhibitor of growth 5, *ING5 (−)* ING5 knockdown, *NC* negative control

**Fig. 8:**
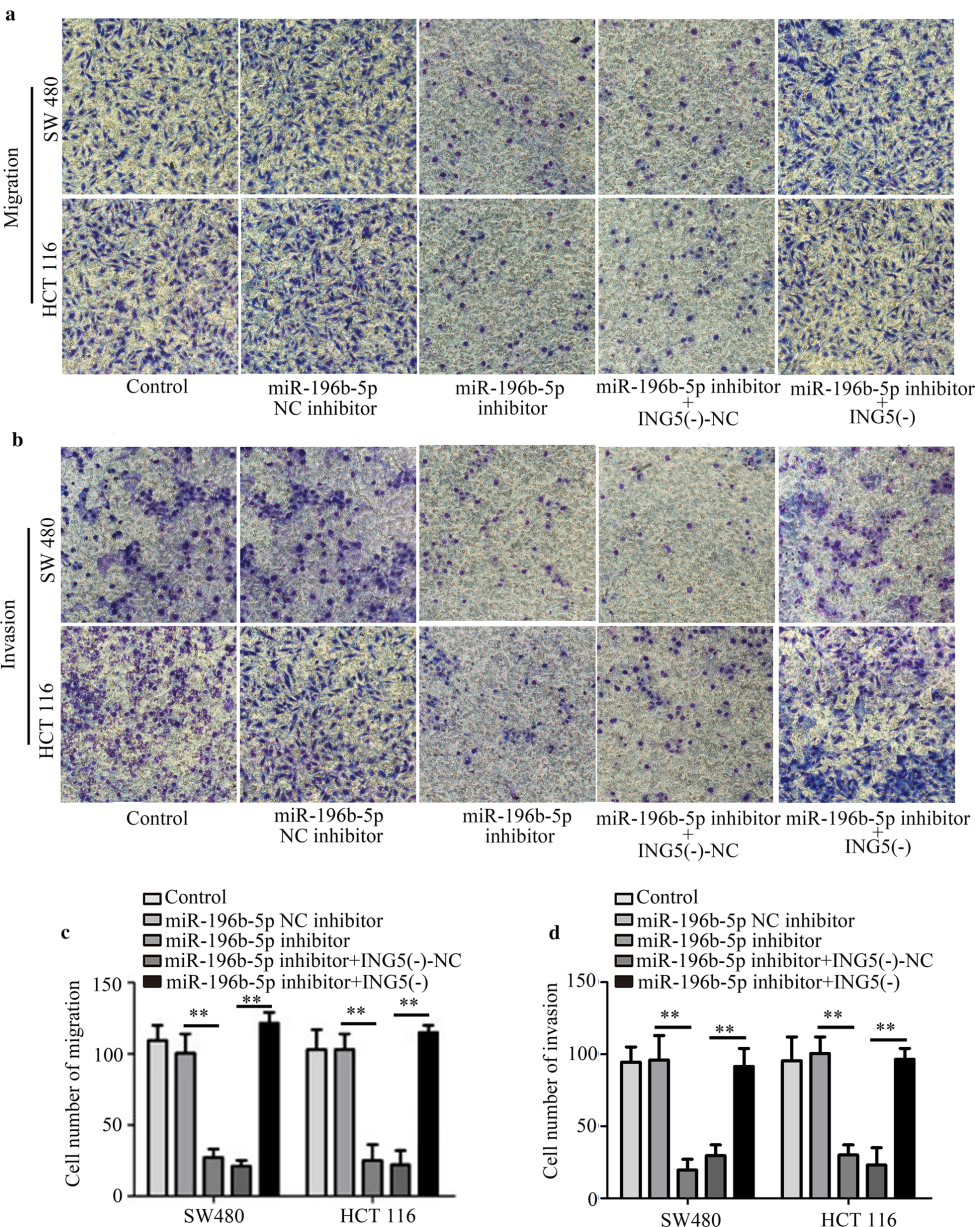
ING5 knockdown mediates the effects of miR-196b-5p inhibitor on CRC cell migration and invasion. **a**, **b** Cell migration and invasion were analyzed through Transwell assays. Scale bar, 20 μm. **c**, **d** Cell migration and invasion were quantified. n = 3. *P < 0.05. **P < 0.01. *CRC* colorectal cancer, *ING5* inhibitor of growth 5, *ING5 (−)* ING5 knockdown, *NC* negative control

**Fig. 9:**
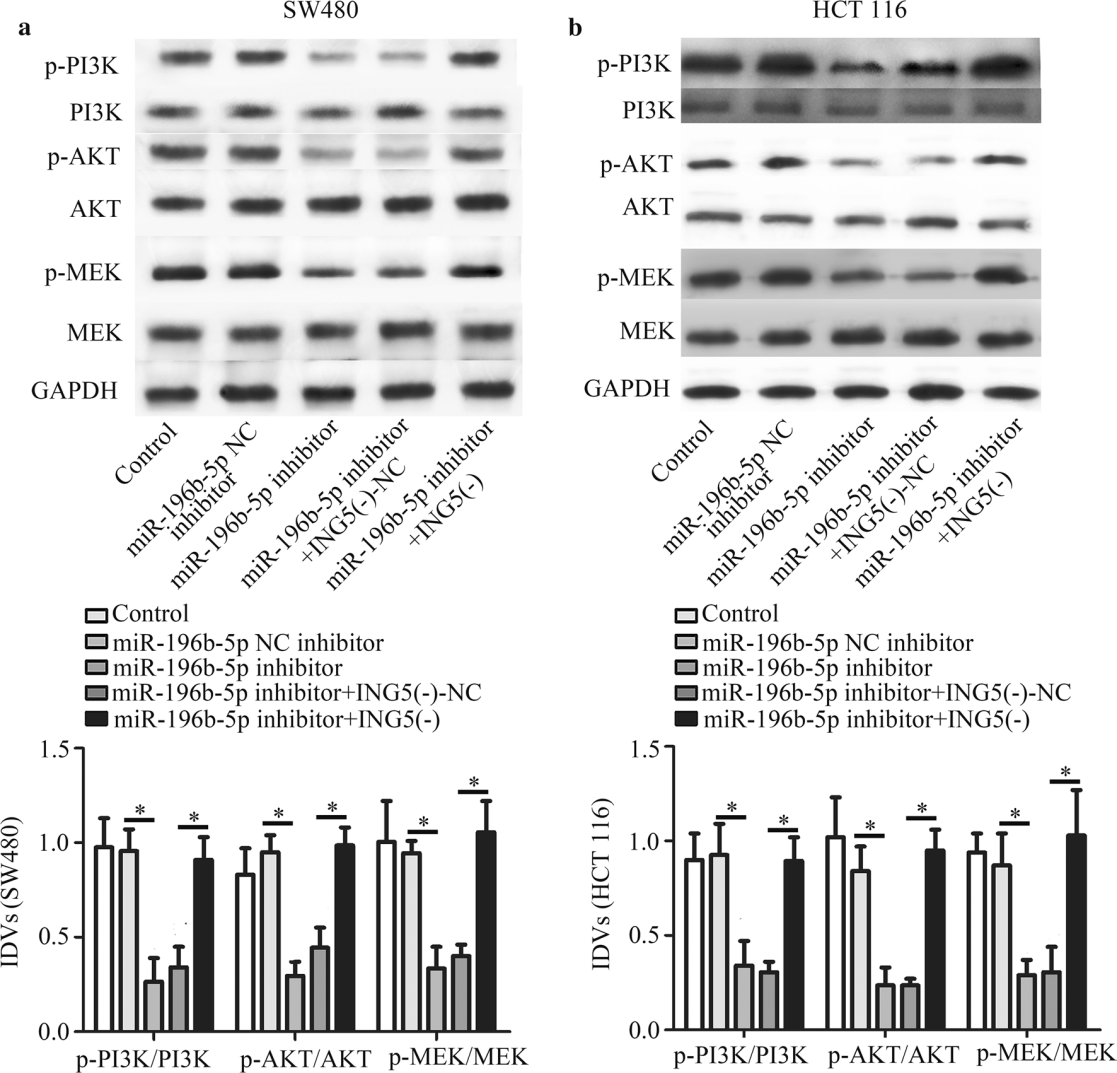
Effects of ING5 knockdown on PI3K/Akt signaling pathway. **a**, **b** The protein levels of PI3K, p-PI3K, Akt, p-Akt, MEK and p-MEK were detected using western blot analysis. n = 3. *P < 0.05. **P < 0.01. *CRC* colorectal cancer, *ING5* inhibitor of growth 5, *ING5 (−)* ING5 knockdown, *NC* negative control

We further confirmed whether the role of miR-196b-5p in CRC progression was through targeting ING5. As shown in Fig. 7a, miR-196b-5p inhibitor treatment inhibited cell proliferation, which could be impeded by ING5 knockdown. Similarly, flow cytometry analysis showed that the apoptosis-inducing effects of miR-196b-5p inhibitor could be suppressed by ING5 knockdown (Fig. 7b). Transwell assays revealed that ING5 knockdown crippled the inhibiting effects of miR-196b-5p inhibitor on migration and invasion (Fig. 8a–d). We further detected p-PI3K, p-Akt, and p-MEK protein levels using western blot analysis. The results showed that miR-196b-5p inhibitor decreased these protein levels, whereas these protein levels were elevated in CRC cells co-transfected with miR-196b-5p inhibitor and ING5 knockdown (Fig. 9a, b). These findings implied that miR-196b-5p was involved in progression of CRC by targeting ING5.

### Effects of miR-196b-5p on tumor growth

**Fig. 10:**
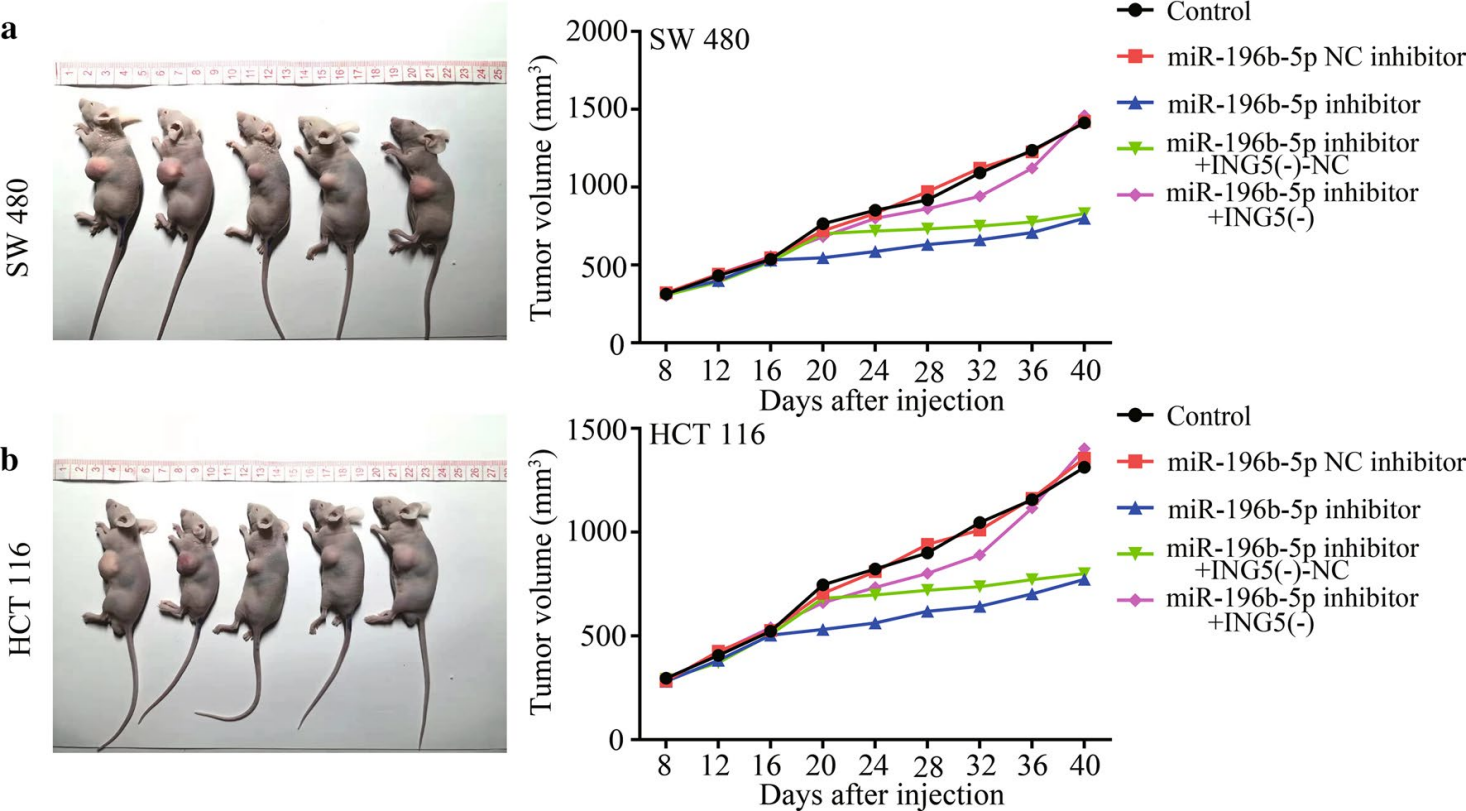
Effects of miR-196b-5p on tumor growth. The mice were subcutaneously injected with 1 × 10^6^ CRC cells. Then **a**, **b** the nude mice carrying tumors from respective groups were shown and tumor volume was calculated every 4 days after injection. n = 3. *CRC* colorectal cancer, *ING5* inhibitor of growth 5, *ING5 (−)* ING5 knockdown, *NC* negative control

The effect of miR-196b-5p on tumor growth was evaluated in BALB/c nude mice. As shown in Fig. 10a, b, the tumor volume was decreased after miR-196b-5p inhibitor compared with the control mice. Moreover, ING5 knockdown facilitated tumor growth, that was, the anti-tumor effect of miR-196b-5p inhibitor could be diminished by ING5 knockdown in vivo. The in vivo results proved that miR-196b-5p acted as a promoter of tumor growth via regulating ING5.

## Discussion

In this study, we mainly investigate the function and potential mechanism of miR-196b-5p/ING5 system on malignant progression in CRC cells, including SW480 and HCT116. Our present results suggested that miR-196b-5p was up-regulated in CRC tissues and cells. In addition, miR-196b-5p promoted proliferation, migration, invasion, and inhibited apoptosis in CRC cells through targeting ING5. For this reason, our results suggested miR-196b-5p was a potential oncogene that regulated proliferation, apoptosis, migration and invasion via targeting ING5. Besides, our study indicated that miR-196b-5p/ING5 signaling could be a therapeutic target candidate for CRC.

Emerging studies have reported that abnormal miR-196b-5p expression was related with the development of various tumors [21, 31–33], whereas the potential molecular mechanisms of miR-196b-5p dysfunction in CRC progression are remained to be revealed. As mentioned in numerous studies, Zhu et al. discovered that miR-196b-5p was decreased in breast cancer, and miR-196b-5p overexpression suppressed tumor growth and metastasis [31]. Xie et al. and Liu et al. found that miR-196b-5p was demonstrated to be reduced in a gastric cancer cell line [32, 33]. However, the effect of miR-196-5p on tumor progression is diverse. Accumulating evidence has showed that miR-196b-5p overexpression is proved to enhance cell proliferation and invasion in gastric cancer [34]. Moreover, Ren et al. reported that miR-196b-5p was significantly increased in CRC tissues, and high miR-196b-5p level showed a correlation with poor survival in CRC patients [21]. In our work, we found that miR-196b-5p up-regulation was verified in CRC tissues and cells. Moreover, miR-196b-5p played an important role in promotion of proliferation, migration, invasion, and suppression of apoptosis in CRC cells, indicating that miR-196b-5p might act as a promoter in CRC progression.

ING5, the last member of Inhibitor of Growth (ING) family, is a well-known tumor suppressor [35, 36]. Reportedly, ING5 was found to repress proliferation, and elevate autophagy and apoptosis in gastric cancer cells [37]. Additionally, ING5 played a suppressive role in proliferation, migration and invasion in lung cancer [38].

Similar to the results of the above research, our results showed that ING5 inhibited cell proliferation, migration, invasion and induced apoptosis in CRC cells. A number of studies indicated that miRNAs could interact with the 3′-UTR of their target mRNAs and took part in the cell process [39, 40]. Previous studies have revealed that miR-1307 participates in ovarian cancer cell proliferation and apoptosis through targeting ING5 [41]. Moreover, miR-27-3p was reported to elevate G1-S transition, causing acceleration of osteosarcoma cell growth via targeting ING5 [42]. This indicates that mRNAs may regulate the cell progression by targeting ING5. Therefore, we then investigated the potential mechanism by which miR-196b-5p promoted progression of CRC, and verified that ING5 was a target of miR-196b-5p. Moreover, ING5 knockdown could elevate the suppression of miR-196b-5p inhibitor in progression of CRC cells in vitro and in vivo. The data indicate that miR-196b-5p may promote cell progression of CRC through targeting ING5.

Emerging evidence indicated that PI3K/Akt signaling related to proliferation and apoptosis, which is often excessive activated in multiple tumors [43]. PI3K/Akt pathway activation was also found to protect cancer cells against apoptosis [44]. Since PI3K/Akt pathway is one of the most common factors associated with cancer progression, PI3K/Akt pathway and the related molecules are regarded as therapeutic targets of cancer treatment [45, 46]. In addition, miR-196b-5p was reported to increase expression of PI3K/AKT/mTOR protein in gastric cancer cells [47]. Meanwhile, ING5 knockdown promoted migration and invasion through up-regulating EGFR/PI3K/Akt signaling pathway in lung cancer cells [43]. On the contrary, ING5 overexpression diminished the ability of proliferation and invasion in hepatocyte growth factor treated thyroid cancer cells via regulation of Akt signaling pathway [36]. Therefore, we wondered whether miR-196b-5p alteration could regulate PI3K/AKT signaling via targeting ING5 expression. Interestingly, our data found that miR-196b-5p inhibitor suppressed the phosphorylation of PI3K, Akt, and MEK, which could be crippled by ING5 knockdown. Hence, we inferred that miR-196b-5p accelerated CRC progression via targeting ING5 and PI3K/Akt signaling pathway. In our present study, we primarily investigated whether miR-196b-5p was involved in CRC progression via targeting ING5. These results implied that miR-196b-5p/ING5 might be an important therapeutic target candidate for CRC treatment. However, there are many target genes of miR-196b-5p, and we will further explore other mechanisms of miR-196b-5p in CRC occurrence and development.

## Conclusion

In conclusion, this study demonstrated that miR-196b-5p promoted the proliferation, migration, invasion and suppressed apoptosis in CRC cells. In contrast, ING5 showed an opposite effects on these cell properties. Moreover, miR-196b-5p could target ING5, and ING5 knockdown could improve the effect of miR-196b-5p inhibitor on cell progression. These results indicate that miR-196b-5p/ING5 signaling might be an effective therapeutic target for CRC.

## Supplementary information

**Additional file 1: Fig. S1.** Effects of miR-196b-5p on miR-196a-1 and miR-196a-2 in CRC cells. (A-D) The levels of miR-196a-1 and miR-196a-2 were detected with quantitative real-time PCR. n = 3. CRC, colorectal cancer. NC, negative control.

**Additional file 2: Fig. S2.** Effects of ING5 on miR-196a-1 and miR-196a-2 in CRC cells. (A-D) Quantitative real-time PCR was used to detect the levels of miR-196a-1 and miR-196a-2. n = 3. CRC, colorectal cancer. NC, negative control.

## Data Availability

We declare that the materials described in the manuscript, including all relevant raw data, will be freely available to any scientist wishing to use them for noncommercial purposes without breaching participant confidentiality.

## Abbreviations

CRC: Colorectal cancer
miRNAs: MicroRNAs
ING5: Inhibitor of growth 5
SPF: Specific pathogen-free
