# Supplementary figures and images for: MicroRNA-196b-5p promotes malignant progression of colorectal cancer by targeting ING5
Source: Cancer Cell Int. 2020 Apr 10;20:119. doi: 10.1186/s12935-020-01200-3 (PMC7149860; doi:10.1186/s12935-020-01200-3)

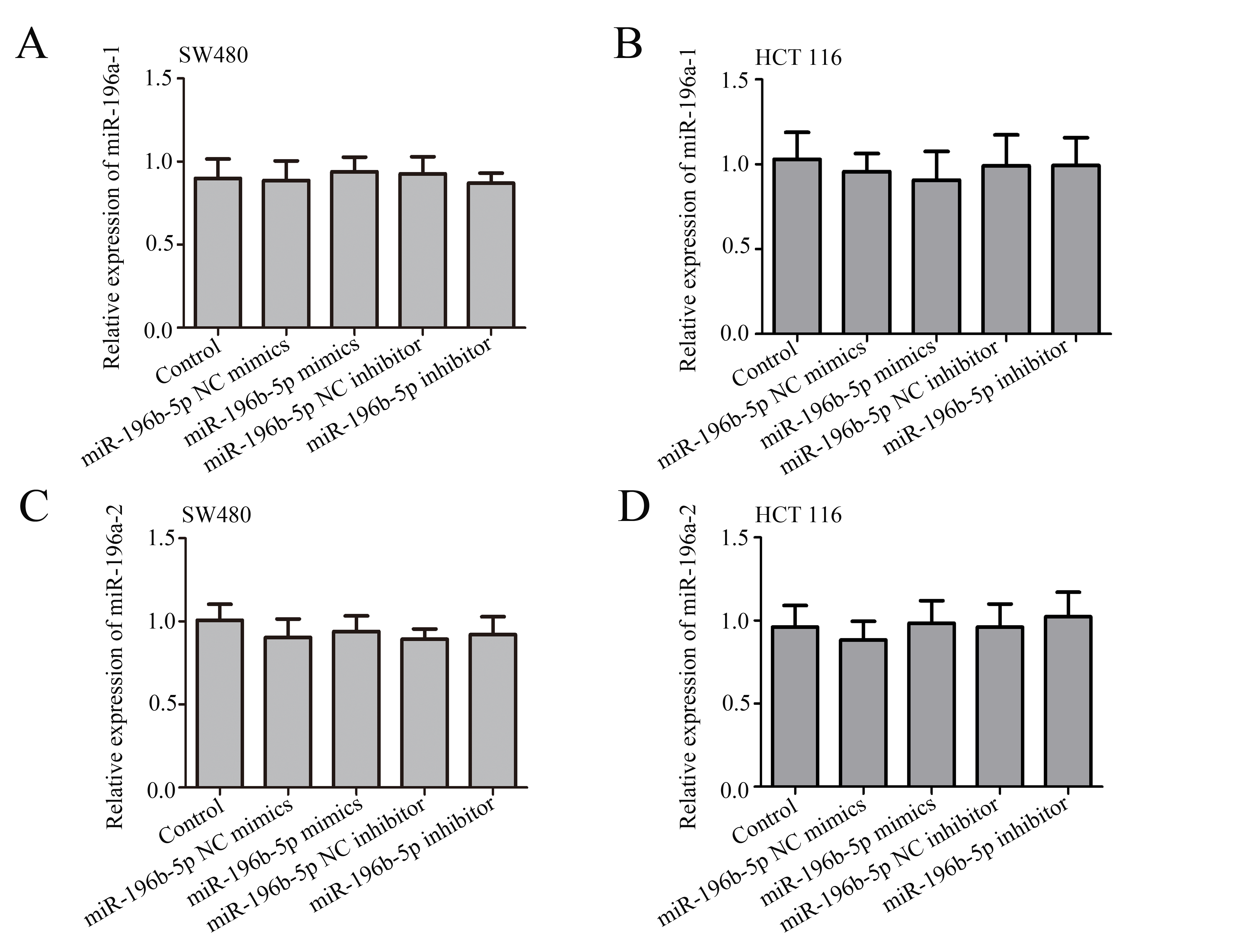

Supplement: Supplementary file 1 — Additional file 1: Fig. S1. Effects of miR-196b-5p on miR-196a-1 and miR-196a-2 in CRC cells. (A-D) The levels of miR-196a-1 and miR-196a-2 were detected with quantitative real-time PCR. n = 3. CRC, colorectal cancer. NC, negative control. [file 12935_2020_1200_MOESM1_ESM.tif]

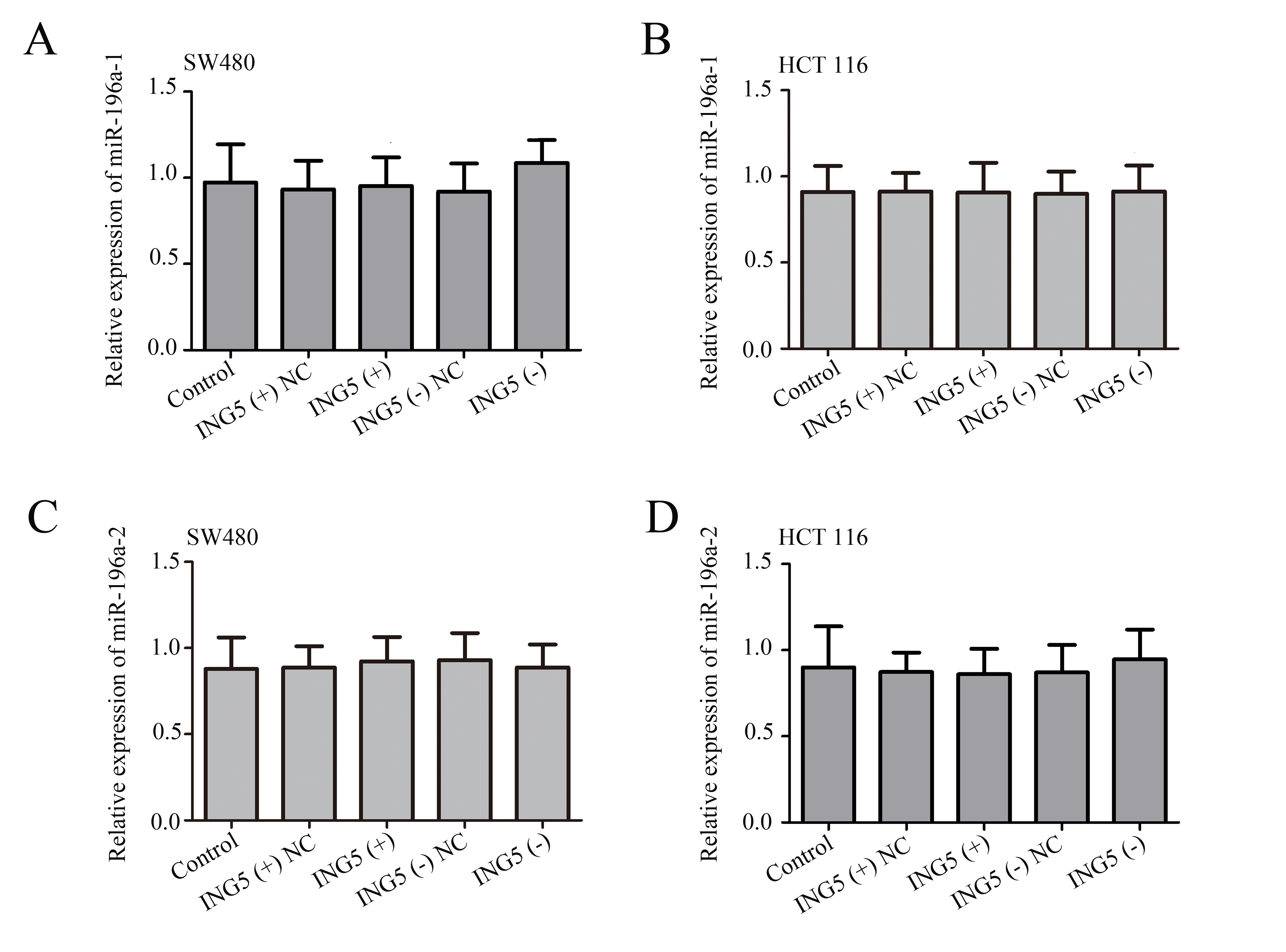

Supplement: Supplementary file 2 — Additional file 2: Fig. S2. Effects of ING5 on miR-196a-1 and miR-196a-2 in CRC cells. (A-D) Quantitative real-time PCR was used to detect the levels of miR-196a-1 and miR-196a-2. n = 3. CRC, colorectal cancer. NC, negative control. [file 12935_2020_1200_MOESM2_ESM.tif]
